# Contemporary ocean warming and freshwater conditions are related to later sea age at maturity in Atlantic salmon spawning in Norwegian rivers

**DOI:** 10.1002/ece3.337

**Published:** 2012-07-28

**Authors:** Jaime Otero, Arne J Jensen, Jan Henning L'Abée-Lund, Nils Chr Stenseth, Geir O Storvik,, Leif Asbjørn Vøllestad

**Affiliations:** 1 Centre for Ecological and Evolutionary Synthesis (CEES), Department of Biology, University of OsloP.O. Box 1066 Blindern, N-0316, Oslo, Norway; 2 Norwegian Institute for Nature ResearchP.O. Box 5685 Sluppen, N-7485, Trondheim, Norway; 3 Norwegian Water Resources and Energy DirectorateP.O. Box 5091 Majorstua, N-0301, Oslo, Norway; 4 Institute of Marine Research, Flødevigen Marine Research StationN-4817, His, Norway; 5 Department of Mathematics, University of OsloP.O. Box 1066 Blindern, N-0316, Oslo, Norway

**Keywords:** discharge, maturation, Norway, *Salmo salar*, sea surface temperature

## Abstract

Atlantic salmon populations are reported to be declining throughout its range, raising major management concerns. Variation in adult fish abundance may be due to variation in survival, growth, and timing of life history decisions. Given the complex life history, utilizing highly divergent habitats, the reasons for declines may be multiple and difficult to disentangle. Using recreational angling data of two sea age groups, one-sea-winter (1SW) and two-sea-winter (2SW) fish originated from the same smolt year class, we show that sea age at maturity of the returns has increased in 59 Norwegian rivers over the cohorts 1991–2005. By means of linear mixed-effects models we found that the proportion of 1SW fish spawning in Norway has decreased concomitant with the increasing sea surface temperature experienced by the fish in autumn during their first year at sea. Furthermore, the decrease in the proportion of 1SW fish was influenced by freshwater conditions as measured by water discharge during summer months 1 year ahead of seaward migration. These results suggest that part of the variability in age at maturity can be explained by the large-scale changes occurring in the north-eastern Atlantic pelagic food web affecting postsmolt growth, and by differences in river conditions influencing presmolt growth rate and later upstream migration.

## Introduction

Recent climate change is promoting multiple effects at population, community, and ecosystem levels inducing extensive ecological changes well documented from various terrestrial, freshwater, and marine systems (Letcher 2009). The impacts of climate variability on aquatic ecosystems are diverse and affect key life history processes, including reproduction and maturation of fish (Rijnsdorp et al. 2009). Age at reproduction is among the most important life history traits, having profound fitness effects and also important demographic implications (Stearns 1992). In fish, age at maturity has a significant additive genetic component (Carlson and Seamons 2008), but is also highly plastic (Hutchings 2011). This is also the case for the Atlantic salmon (*Salmo salar*) (García de Leániz et al. 2007), a highly charismatic species with great economic and social value. It is therefore of great concern that Atlantic salmon production has been declining across most of the species’ distributional range over the past recent decades (Hindar et al. 2011).

The life history of Atlantic salmon is complex (Thorstad et al. 2011). Spawning occurs in freshwater in October–January. After hatching in spring the juveniles (parr) stay in freshwater 1–6 years before transforming into smolt. The smolts then leave their rivers to pursue oceanic feeding migrations during spring and early summer. Post-smolt Atlantic salmon spend 1–4 years at sea until the attainment of sexual maturity, and return in May–October with high natal fidelity to spawn. Maturation may be reached after a single (1SW) or multiple winters at sea (MSW) and timing of maturation is to some extent genetically determined (García de Leániz et al. 2007). Although some results indicated that sea age at maturation is fixed when the smolts leave their rivers (Chadwick et al. 1987), the biochemical processes leading to the completion of maturation are complex and depend on environmental conditions. Hence, the reproductive strategy might be environmentally dependent, but genetically determined through a threshold level that prevent or permit an individual to continue further gonadal development at a critical decision time (Thorpe 1994). Several studies have shown that whether to continue maturation or not is determined during autumn, a year ahead of spawning (Jonsson and Jonsson 2011). If the level of energy stores has not reached a genetically specified threshold level completion of maturation is halted (Mangel and Satterthwaite 2008). This arrest suggests that the proportion of fish maturing at a given sea age would vary from year-to-year as a function of variability in feeding conditions. On top of this there might be differences among populations in thresholds and reaction norms (Piché et al. 2008).

Previous studies have shown that the year-to-year variability in the proportion of salmon maturing as 1SW relative to MSW (often called the grilse to salmon ratio) is associated with changes in sea temperature. Warmer temperature was reported to result in an increased number of fish returning as multi-sea-winter salmon to Scottish (Martin and Mitchell 1985) and Icelandic (Gudjonsson et al. 1995) rivers. However, the nature and mechanisms of the environmental effects across life stages both in the ocean and in the river that in turn might affect the age at maturity are still unclear. During the parr stage, juveniles may be affected by water flow (e.g., Jensen and Johnsen 1999) and river temperature (e.g., Jensen 2003). Freshwater conditions influence presmolt growth rates that, in turn, might be associated with growth during the first year at sea and hence sea age at maturity. If so, age at maturity may partly be determined prior to sea entry. Some studies indicate that smolt size and marine growth are positively related (e.g., Salminen 1997), whereas other authors have found an inverse relationship (e.g., Nicieza and Braña 1993). In addition, some studies suggest that increased growth rate during the first year at sea leads to postponement of maturation (e.g., Jonsson et al. 2003), whereas other studies argue the opposite (e.g., Friedland and Haas 1996).

In this study we used time series of the proportion of one-sea-winter (1SW) relative to two-sea-winter (2SW) fish from the same smolt year class of Atlantic salmon angled in 59 Norwegian rivers to study the year-to-year variability in age at maturation. We first examined the time trend of one-sea-winter proportions, and then we hypothesized that (i) the oceanic conditions experienced by postsmolts in their marine foraging habitat, and (ii) the environmental conditions during the presmolt freshwater residence previous to the seaward migration, affect the annual returning proportion of the two sea age groups of Atlantic salmon.

## Material and methods

### Biological data

The present study is based on the official statistics of nominal rod catch of adult Atlantic salmon for the period 1992–2007 over a wide geographical range of Norwegian rivers (58°19′–70°37′N and 5°07′–30°32′E). The legal fishing season is restricted to summer and early autumn (mainly June–August), but differs somewhat among rivers. However, the fishing season within each river was constant during the study period. Fishing effort in Norwegian rivers has not been recorded during the study period. There could be some among year variation in effort, induced by environmental variation, such as differences in weather conditions and water flow. We are not able to adjust for such variation due to lack of information, and this will introduce more noise into our analyses (see Otero et al. 2011 for a discussion). In Norway, systematic collection of data on salmonid fisheries began in 1876. Starting in 1979, Atlantic salmon were identified at the species level and differentiated into two weight categories (<3 kg and ≥3 kg). Sex is not recorded in the statistics. The smaller group mainly corresponds to 1SW fish (grilse), and the larger group corresponds to MSW fish. Starting in 1993, the MSW group was categorized into two more weight classes (3–7 kg and ≥7 kg) corresponding to 2SW and mainly 3SW fish, respectively (Jensen et al. 1999). This categorization into sea age classes based on their weight as reported by anglers might potentially introduce a bias if the binning is incorrect or varying with time or among rivers. We have obtained estimates of sea age based on scale analysis from 27 rivers, all of them included in this study (4062 samples, variation 91–437 among rivers). These data showed that 93.8% of 1SW fish were < 3 kg. Furthermore, 80.8% of 2SW fish were between 3 and 7 kg, and 87.8% of 3SW or older fish were ≥7 kg (Fig. S1). Hence, based on our observations showing that only a small fraction of fish was misclassified a potential bias seems minor (see also Borgstrøm et al. 2010). Furthermore, there does not seem to be significant temporal trends in the bias during the studied period (Arne J. Jensen, unpublished data), indicating that the classification is robust.

For the purpose of this study we used data from the 1SW (i.e., the number of fish from years 1992 to 2006) and 2SW (i.e., the number of fish from years 1993 to 2007) weight groups compiled from 59 Norwegian rivers (Table S1). We based our analyses on the annual proportion of angled fish attaining maturity as 1SW fish relative to 1SW and 2SW fish pertaining to the same smolt cohort, i.e., 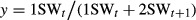, over the cohorts 1991–2005 (Fig. S2). Fish with higher sea age were discarded as they have experienced additional environmental constraints in the freshwater and/or ocean habitat. Further detailed description of data handling and discussion on possible uncertainties, as those ascribed to the use of rod catches as surrogates for population abundances, can be found elsewhere (Otero et al. 2011).

### Environmental data

Long-term changes in the sea surface temperature (SST, °C) have strong impacts on the North Atlantic Ocean ecosystem (e.g., Beaugrand 2009), and have been associated with variation in Atlantic salmon growth rates (Todd et al. 2008) and catch (Beaugrand and Reid 2003). Therefore, we used basin scale SST as a surrogate of oceanic conditions. Optimum Interpolation sea surface temperature (SSTv2) data available at 1° latitude × 1° longitude grid resolution were obtained from the NOAA Earth System Research Laboratory (http://www.esrl.noaa.gov/psd/). Monthly average data from a combination of satellite and in situ measurements (Reynolds et al. 2002) were extracted for the period May-1991 to April-2006 to match appropriate time lags (see below), and delimited to the range of 55°–80°N and 15°W–30°E (note that the Baltic Sea was excluded from the analyses). This area covers most of the migratory and foraging habitat of Atlantic salmon originated from Norwegian rivers (Holm et al. 2004). Different months were tried as covariates (see below). Because each time series has a spatial coverage, monthly SST was analyzed using Principal Component Analysis (PCA). Thus, a separate PCA was then performed for each monthly time series (i.e., each of the 12 matrices comprised 15 years × *p* grid boxes, where *p* was the number of grid boxes that varied among months depending on the affection by sea ice) to identify long-term changes and large spatial scale patterns in SST.

Water discharge was used as a surrogate of freshwater conditions in terms of feeding opportunities and growth rates. Mediated, for instance, by river temperature (Forseth et al. 2001), variation in discharge might cause differences in growth and other traits within and among populations (Jensen and Johnsen 1999; L'Abée-Lund et al. 2004) River conditions in midsummer may determine whether an individual will undergo smolt transformation and emigrate from the river the following spring (Mangel and Satterthwaite 2008), and also the size of those that do migrate. Therefore, we considered average discharge in each river during spring-summer (May–August) one-year ahead of smolt migration for the period 1990–2004 as a potential predictor. At present more than 550 gauging stations covering most of the larger rivers in Norway measure discharge at least once a day. To adjust all the discharges to the river mouth and to produce daily discharge for those rivers without any hydrological monitoring, daily discharge (m^3^/s) for each river catchment was estimated using a spatially distributed version of the Hydrologiska Byråns Vattenbalansavdelning model (HBV, http://www.smhi.se/foretag/m/hbv_demo/html/welcome.html) developed by the water balance section of the Swedish Meteorological and Hydrological Institute (see Beldring et al. 2003; Otero et al. 2011).

### Statistical analyses

First, we fitted a linear regression to each time series of 1SW fish proportion and related the individual time slopes to a set of river characteristics (L'Abée-Lund et al. 2004). Second, we fitted a population model to all time series using linear mixed-effects models (random grouping factor comprises 59 rivers with 867 observations) following methods described in Pinheiro and Bates (2000). Third, we modeled the time series as a function of the environmental factors. In all analyses the response variable (i.e., the proportion, *y*, derived from the counts) was logit transformed (i.e., 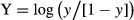, there were not proportions equal to 0 and 1 that needed further solution). Parameters were estimated using restricted maximum likelihood (REML), and selection of explanatory variables, random effects, and correlation structure on error term was all performed using the Bayesian Information Criterion (BIC) that puts a heavier penalty on models with more parameters. A preliminary analysis indicated that inclusion of a random effect in the intercept might be required to account for river-to-river variability (Table S2). According to these preliminary results we fitted a model of the form:


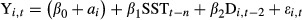
(1)

where Y is the logit-transformed proportion of 1SW fish for each river *i* angled during the fishing season in year *t*; SST is the first principal component of the SST analyses lagged depending on the considered month, that is, SST from May to December was lagged one year (i.e., first to eighth month at sea, representing temperature effects during the smolt year, subscript *n* = 1), and months from January to April were not lagged (i.e., 9th to 12th months at sea, representing temperature effects during the returning year for 1SW individuals, subscript *n* = 0); and D is ln-transformed discharge with a 2-year lag to represent effects during presmolt growth conditions the year before emigrating from the river. Note also that D was centered subtracting the whole population of river's ln-mean. *β*_j_ are the fixed effects to be estimated; *a* is the random river (*i*) effect for the intercept assumed to follow a normal distribution with mean zero and variance 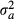; and *ɛ*_*i*,*t*_ is the within-group error term. Different autoregressive moving average (ARMA) structures were tested to model within-group serial correlation in *ɛ*_*i*,*t*_ (Table S2). BIC indicated that an AR model of order 1 [AR(1)] provided the better fit of the data (i.e., *ɛ*_*i*,*t*_ = *φɛ*_*i*,*t*-1_ + *η*_*i*,*t*_, where 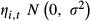). Furthermore, the variance in residual proportion was modeled as an exponential function of the fitted values (i.e., 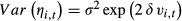, where *δ* is a parameter to be estimated that describes the estimated change in variance with the fitted values (*v*_*i*,*t*_)). All analyses were performed on R 2.15.0 language (R Core Development Team 2012) and using the “nlme 3.1–103” package (Pinheiro and Bates 2000).

## Results

**Figure 1:**
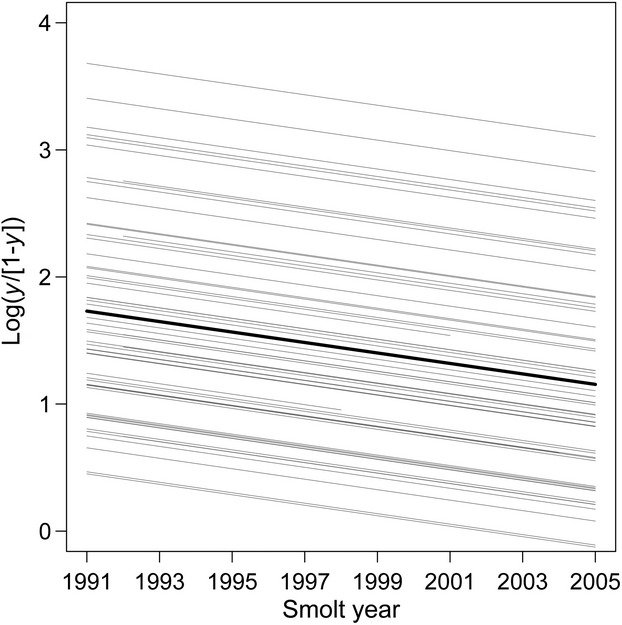
Predicted values obtained by fitting a random intercept model to the proportions’ time series. The black line represents the fitted values for the population of rivers and is specified by the following equation 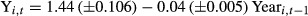 where both coefficients are statistically significant (*P* < 0.0001). Whereas the gray lines represent the within-group fitted curves (see further details in Fig. S2).

There was a large temporal and spatial variation in the proportion of 1SW Atlantic salmon from the 1991–2005 cohorts that were captured in Norwegian rivers during the 1992–2007 fishing seasons. The proportion of 1SW Atlantic salmon during the smolt year classes from 1991 to 2005 generally decreased with time across the rivers studied (Fig. S2), and the estimated individual long-term trends were not related with latitude or any of a set of river characteristics (Fig. S3). Overall, the predicted odds of 1SW fish for the whole population of rivers decreased by a factor of 0.96 per year (i.e., a 4%/y) (Fig. 1, Fig. S2).

**Figure 2:**
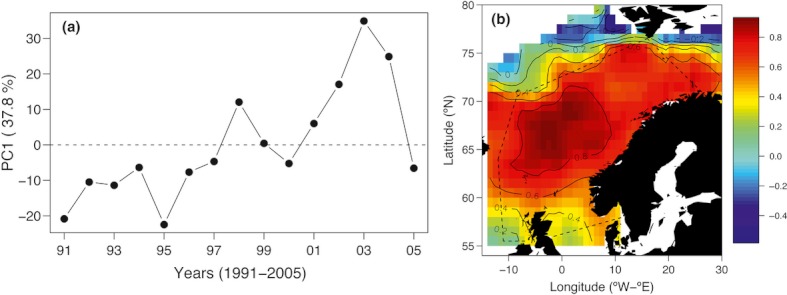
Large spatial patterns in SST in the Norwegian Sea. (a) First principal component of SST in September over the period 1991–2005 accounting for 37.8% of the total variance. (b) Spatial contour plot for the correlations (loadings) of the first principal component for SST in September. The dashed line delimits the approximate area of distribution of postsmolt Atlantic salmon according to Holm et al. (2004). Note that the Baltic Sea data were excluded from the analysis. Northernmost white areas were affected by sea ice and not included in the analysis.

**Figure 3:**
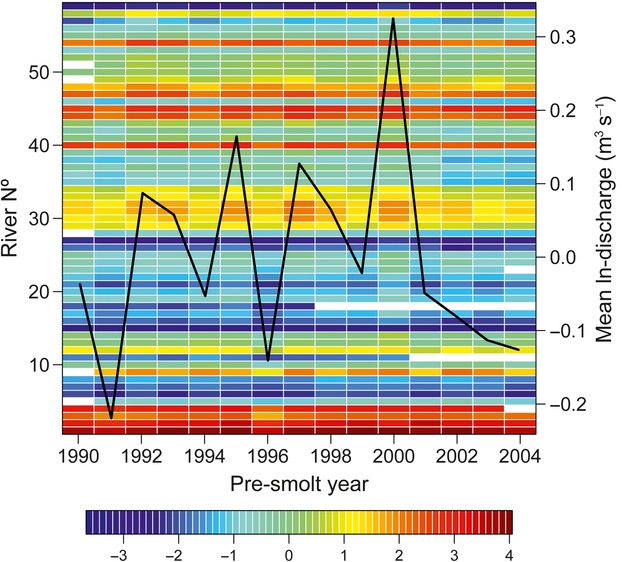
Estimated ln-transformed discharge (m^3^/s) from May to August in the year prior to Atlantic salmon smolt transformation during the years 1990–2004 for each river 1–59. Discharge has been centered by subtracting the whole population of river's ln-mean (3.36 m^3^/s). The black line shows the overall annual mean.

Regarding the environmental factors, the first principal component of September SST in the North-east Atlantic, that is the month that gave the better fit in the mixed-effects model in terms of the BIC criteria (Table S3), accounted for 37.8% of the total variance and showed an increasing trend over the time period investigated (Fig. 2a). This warming was spatially structured with the grid cells with higher correlations centered in the central Norwegian Sea and north of the Faroe Islands (Fig. 2b). This region corresponds to the general area of oceanic distribution for Atlantic salmon postsmolt. By contrast, river discharge was relatively stable across years within each river (Fig. 3). Overall, the most remarkable feature occurred in year 2000 with an apparent increase in the average discharge.

**Figure 4:**
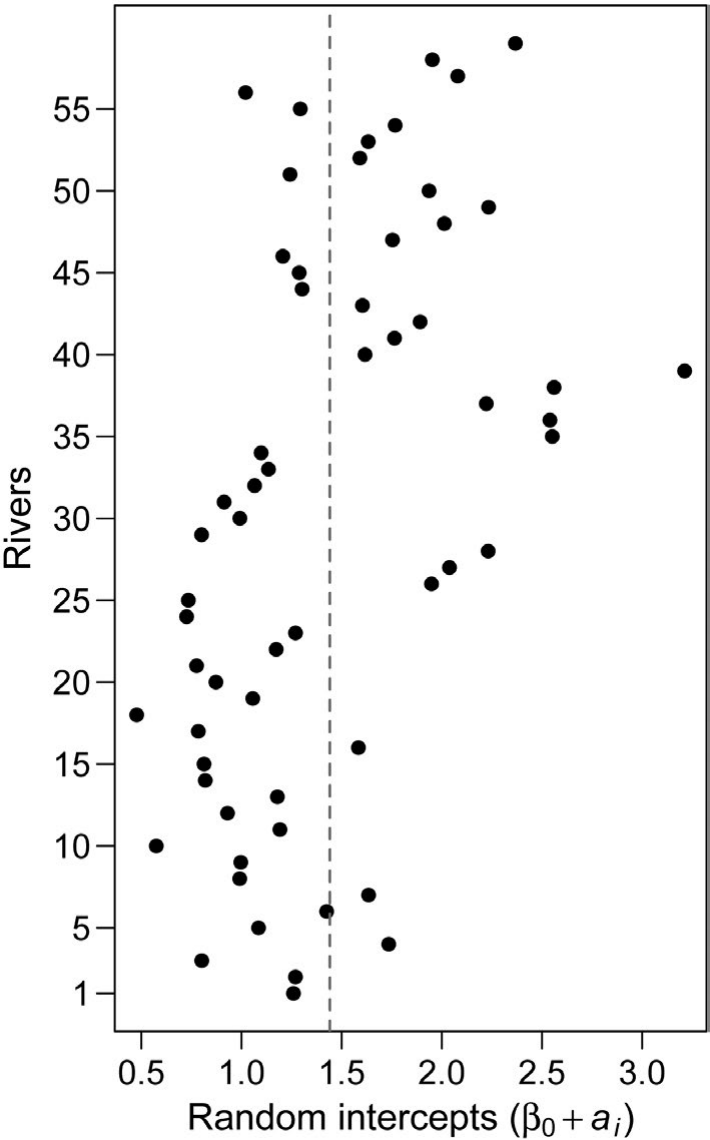
Estimated random intercepts for rivers. River-specific intercepts (*β*_0_ + *a*_*i*_) representing the predicted logit-transformed 1SW fish proportion at a value of zero of the PC1 of SST in September and at the ln-mean value of discharge. The gray dashed line represents *β*_0_ (Table 1). The rivers are numbered chronologically along the Norwegian coast from south to north. See Table S1 for rivers’ numbers.

The optimal mixed-effects model showed that warmer ocean temperatures in autumn (September) were associated with a decrease in the proportion of 1SW Atlantic salmon (Table 1, Fig. S4a). Moreover, higher discharge in summer months 1 year before smolt transformation was also related with a decrease in the proportion of 1SW fish for the period 1991–2005 (Table 1, Fig. S4b). Estimated random intercepts for rivers showed a latitudinal gradient as indicated by an increase in the predicted proportion of 1SW fish from southern to northern rivers (Fig. 4). The lag 1 coefficient (*φ*) for the autocorrelation structure was negative suggesting that age groups from the same cohort were inversely related. Furthermore, the estimated parameter (*δ*) of the variance function was positive, indicating higher variability at larger fitted values (Table 1). Within-group residuals from the optimal model were normally distributed and did not show any remaining variability. Random effects were also reasonably normally distributed (Fig. S5).

**Table 1:** Optimal environmental model results. Results from analyses of the influence of SST (PC1) and discharge on the logit-transformed proportion of one-sea-winter relative to two-sea-winter fish from the same smolt cohort of Atlantic salmon obtained from the optimal mixed-effects model with River as random grouping factor (59 levels)

Effects	Estimate	95% CI	*t*-value	*P*-value
Fixed				
Intercept	1.441	1.279; 1.602	17.509	<0.0001
SST_*September*_	–0.012	–0.014; –0.009	–9.257	<0.0001
D	–0.289	–0.378; –0.200	–6.370	<0.0001
Random (SD)				
Intercept ( 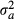 )	0.612	0.501; 0.747	na	na
Residual (*σ*^2^)	0.496	0.446; 0.550	na	na
Correlation structure				
*φ*	–0.140	–0.212; –0.067	na	na
Variance function				
*δ*	0.214	0.150; 0.278	na	na

SST = sea surface temperature, D = discharge, CI= confidence interval, SD = standard deviation, na = not applicable.

## Discussion

Using data on Atlantic salmon catch statistics for 59 Norwegian rivers we showed that sea age at maturity in Atlantic salmon, measured as the proportion of 1SW–2SW fish originating from the same smolt year class, has increased over the cohorts 1991–2005 and appeared to be influenced by environmental conditions in the ocean during the postsmolt phase (early autumn) as well as in the river during the parr stage 1 year before smolt migration.

**Figure 5:**
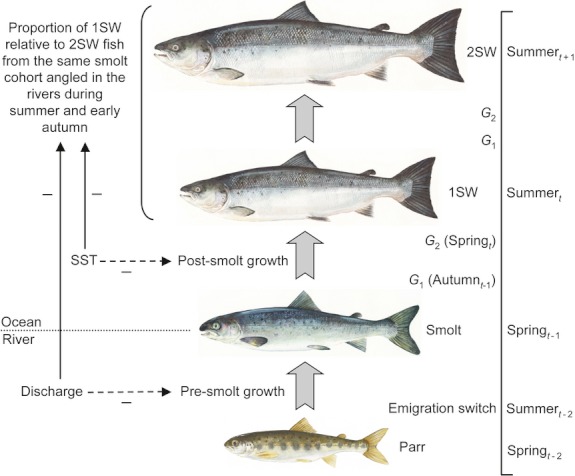
Schematic representation of the Atlantic salmon life cycle and the relationships reported in this study. Fish mature after one-sea-winter (1SW) or two-sea-winters (2SW) depending on the route determined by the responses to the developmental switches *G*_1_ and *G*_2_ (see Mangel and Satterthwaite 2008). Black arrows indicate the (negative) relationships modeled in this work, whereas dashed arrows show the hypothetical (negative) effects of sea surface temperature (SST) and discharge on post- and presmolt growth, respectively. See the main text for further discussion on the potential nature of the relationships involved. The modeling framework by Thorpe et al. (1998) inspired this sketch. Drawings credits: © Atlantic Salmon Federation (http://www.asf.ca)/J.O. Pennanen.

To complete the maturation process and be ready to reproduce in autumn, Atlantic salmon must initiate physiological changes well in advance. Mangel and Satterthwaite (2008), building on work by Thorpe (see Thorpe et al. 1998), summarized a life history framework describing developmental switches that occur in the fish at a given decision time (a “window of opportunity”, Jonsson and Jonsson 2011) (Fig. 5). That is, in autumn, if the lipid levels and the rate of their change with respect to a genetically determined threshold (switch designated as *G*_1_) are reached, gonad development continues and the fish will mature; otherwise maturation is inhibited. The following spring a similar comparison between the state of lipids and their rate of change with a second threshold occurs (switch designated as *G*_2_). Therefore, 1SW fish have followed a path where the thresholds at *G*_1_ and *G*_2_ were reached, whereas 2SW fish have followed a path where either the threshold at *G*_1_ was not reached or it was reached at *G*_1_ but not at *G*_2_. Overall, our results show that, over the 15-year period examined, more salmon in Norwegian rivers are maturing as 2SW fish, meaning that the proportion of Atlantic salmon from a given cohort that attains maturity after 1 year at sea is decreasing. The predicted 1SW proportion, however, showed spatial heterogeneity with higher values in northern rivers (Fig. 4) indicating that despite the average decreasing trend in 1SW fish proportion, there was a latitudinal gradient of increasing importance of this age group from southern to northern rivers. This spatial variation could be attributed to other environmental and/or biotic components not included in our study (L'Abée-Lund et al. 2004). Concurrent with this result, Otero et al. (2011) found increasing trends in 1SW abundance in northern Norway. Furthermore, the increase in variance with fitted values could be interpreted as an increase in instability in higher catches of 1SW.

The control of maturation of Atlantic salmon has been tested in aquaculture experiments providing strong evidence that gonad development is halted by poor feeding opportunities and concluding that the amount of lipid stores would probably be the resource governing the direction of maturity at the critical decision time (see Thorpe et al. 1998 and references therein). Low levels of stored lipids would cause maturation to be postponed. Unfavorable feeding conditions at sea may therefore lead to a higher proportion of Atlantic salmon arresting maturity. In this sense, we found that the lower proportion of 1SW fish spawning in Norwegian rivers was associated with warmer SST during September the year before, suggesting that this effect is related to the temperature-induced changes in the food web structure. There are well-described changes in the eastern North Atlantic food web, specifically related with the changes in the zooplankton assemblages occurring as a consequence of ocean warming (Beaugrand 2009). That is, the quantity and quality of prey items would compromise the acquisition of sufficient energy to reach the lipid threshold needed to complete maturation. In detail, the increase in temperature has profound effects on the food web (plankton) composition, particularly leading to a decreased copepod size (Beaugrand 2009). This change in species and size structure could lead to a reduced concentration of lipids available to fish (e.g., Pepin and Head 2009). Copepods (*Calanus* spp.) are the most important constituents of the pelagic ecosystem and serve as food for organisms at higher trophic levels. Even if *Calanus* spp. are not a significant prey item for the Atlantic salmon postsmolt itself (Jacobsen and Hansen 2001), organisms directly dependent on them including various salmon prey items important for the postsmolt, for instance, amphipods, lantern fishes, pearlsides, euphausiids, shrimps, or herring (Jacobsen and Hansen 2001; Haugland et al. 2006), which are decisive for energy – lipid – flux (Jensen et al. 2007; Petursdottir et al. 2008). The effects of climate variation on biochemical ecology are, however, poorly known, although significant variability in lipid content is expected following changes in the availability of essential fatty acids due to changing physical oceanography (e.g., Litzow et al. 2006). Model selection suggested SST effects in September as the most optimal fit (fifth month at sea indicating that the threshold at *G*_1_ was not reached), but note that April might be also an important month (Table S3) (12th month at sea that would indicate that the threshold at *G*_2_ was not reached). This result fits well with the Mangel and Satterthwaite (2008) model, which suggests an additional sensitive period during late winter.

The content of stored lipids in returned 1SW salmon strongly decreases with poorer postsmolt growth condition (Todd et al. 2008) that, in turn, is associated with positive anomalies of SST (Friedland et al. 2005; Todd et al. 2008; but see Bacon et al. 2009). Thus, it seems plausible that lower postsmolt growth mediated by warmer SST leads to an increase in age at maturation. However, there are apparent discrepancies between studies when describing relationships across life-stages and the implication of environmental factors. Studies within (Jonsson et al. 2003) and among Atlantic salmon populations (Hutchings and Jones 1998) have shown that higher growth rate at sea seems linked to an increase in sea age at maturity. This model is in opposition to the one presented by Mangel and Satterthwaite (2008) and to the more general observations based on theory and data that life history transitions happen later when growth rate decreases (e.g., Day and Rowe 2002). A number of other studies in Atlantic salmon, however, support our conclusions. For instance, Friedland and Haas (1996) found a positive relationship between growth during late summer and the fraction of postsmolt attaining maturity after 1 year at sea. Moreover, Salminen (1997) reported a negative relationship between marine growth rate and sea age at first maturity. Furthermore, Jonsson and Jonsson (2004) showed that the percentage of adults maturing as 1SW decrease at lower values of the North Atlantic Oscillation (NAO) from February to April. The SST variability associated with the winter NAO shows marked spatial structure with positive anomalies at lower values of NAO in the postsmolt foraging habitat (Hurrell and Dickson 2004). Besides, postsmolt growth in summer months (fourth and fifth month at sea) drives survival (McCarthy et al. 2008), and survival decreases with warmer SST in the postsmolt foraging area during the same period (Friedland et al. 2009). Postsmolt growth and survival for different sea age groups are strongly correlated, and determined early in the first months of life in the ocean (Jensen et al. 2011). However, we cannot rule out completely that an increase in survival in the second year at sea relative to the first year can contribute to the observed trends in the proportion. Different survival could be a result of different age classes of salmon using different feeding areas (Holm et al. 2003; Dadswell et al. 2010). As changes in SST are varying in intensity across the Atlantic, such divergent habitat use may lead to a temporal trend in the proportions of the different age classes in the catch. Moreover, Martin and Mitchell (1985) hypothesized that the association between a decrease in the proportion of 1SW returns and higher values of sea temperature was related to the possibility of migrating further north in warmer years. A longer migration may indeed incur higher costs.

Age at maturity may partly be determined prior to sea entry. In line with this, we found that the proportion of 1SW fish decreases when river discharge during summer (May to August) before smolt migration increases. This is assumed to be the time when the decision whether or not to emigrate the following spring is made (Thorpe et al. 1998). Experimental studies have shown lower growth rate and lipid content of parr at higher discharge, suggesting that the energetic costs for foraging in response to increased discharge and water velocity are high enough to reduce performance (Kemp et al. 2006). Moreover, field observations revealed lower growth of salmon parr in years with high discharge attributed to a washout or mortality of insect larvae prey during spring floods (Jensen and Johnsen 1999). Therefore, it is plausible that elevated discharge could lead to poor growth and smaller smolt size. Some studies do indicate that smolt size, or related traits, are linked to age at maturation. For example, Chadwick et al. (1987) found an inverse correlation between ovarian development and sea age at maturation, indicating that smolts are already “programmed” for a certain sea age at maturity. However, correlation analyses between stage-specific traits (presmolt growth, smolt size and age, postsmolt growth, and age at maturation) seem to be again in disagreement. For instance, it has been shown that smolt size and marine growth could be positively (e.g., Salminen 1997) or negatively related (e.g., Nicieza and Braña 1993). In addition, both studies found that smaller smolts (slower growth in the river) would mature at older (sea) ages. On the other hand, presmolt growth in freshwater might be negatively linked with growth at sea (e.g., Einum et al. 2002), whereas Friedland et al. (2006) provided no evidence for a relationship among growth rates between life stages. Furthermore, among-population studies have shown that the smaller the fish at seaward migration (salmon growing poorly in freshwater), the higher the subsequent growth at sea (Jonsson and Jonsson 2007). In total, all these apparent discrepancies among correlational studies between stage-specific traits might be related to the exhibition of different reaction norms for growth among populations (Jonsson and Jonsson 2011). Besides this reasoning, other studies already identified a negative association between grilse proportion and discharge suggesting a selective impact of water flow on adult body size mediated by successful ascent of larger fish in rivers with higher flow (e.g., L'Abée-Lund et al. 2004; Power 1981). Both arguments, lower growth at higher discharge and adult fish size related to water level, wouldn't be mutually exclusive.

In contrast to females, male Atlantic salmon may mature as precocious parr (Fleming 1996). The probability of reaching maturity as parr is context dependent, varying with density and growth opportunity in freshwater (Gross 1985; Hutchings and Myers 1994). If the incidence of parr maturation increases with time, this will lead to a change in the sex distribution of the migratory part of the population. One possible consequence of a higher proportion of females is that they will bias the population estimate, as females tend to mature at a higher sea age than males (Fleming 1998). We are aware of one Norwegian river where data are available for both the frequency of precocious males and the number of 1SW and 2SW fish. In this river, Orkla in Central Norway, there is no significant correlation between the frequency of precocious male and sea age at maturity (*n* = 8, *r*^2^ < 0.001, *P* >> 0.1; Arne J. Jensen, unpublished data).

Changes in age and size at maturity in exploited fish could, however, be an evolutionary consequence of continuous fishing (Jørgensen et al. 2007). Selective exploitation can cause detectable evolutionary changes in multiple life traits, but separating phenotypic plasticity from genetically based responses is still complex (Allendorf and Hard 2009). For instance, Fukuwaka and Morita (2008) found that the cessation of high seas fishing activities seemed to be responsible for the observed changes in size at maturity in chum salmon *Oncorhynchus keta*. In line with this, the Norwegian drift net fishery was closed in 1989 leading to demographic changes in populations from Norwegian and Russian rivers (Jensen et al. 1999). However, evolutionary effects for sea age at maturity are less likely due to the fact that this fishery – and the recreational fishery examined here – targeted the spawning run, that is, individuals that have already made the decision to mature. Therefore, it seems that environmental effects would be more important than evolutionary effects. However, we cannot rule out completely the hypothesis of evolutionary effects of fishing derived from responses to the fishery in the foraging area to the north of the Faroe Islands.

To conclude, the abundance of 1SW Atlantic salmon angled in Norwegian rivers increases with coastal temperatures at the time of leaving the freshwater habitat (Otero et al. 2011), however, it seems that poorer feeding conditions at the open ocean in recent years might affect postsmolt growth and thus timing of maturation. Our finding that the proportion of 1SW over a 15-year period has decreased associated with warmer conditions concurs with similar results of various Pacific salmon species where the changes in size and age at maturity was attributed to a plastic response following environmentally induced reduced growth rates (see Morita and Fukuwaka 2007 and references therein). It is therefore likely that the process of later maturation will continue as projected effects of climate change predict a continuous rise of SST, and consequently alterations in the pelagic ecosystem of the eastern North Atlantic Ocean and adjacent seas (Beaugrand 2009). Furthermore, increased water discharge at the presmolt stage may promote poor growth during freshwater residence, although the (in)direct effects on posterior oceanic growth and later on the sea age at maturity are still unclear. Climate models point toward an increase in precipitation and springtime flooding events (Benestad and Haugen 2007), thus it is expected that these conditions would potentially affect growth rate in freshwater. Therefore, it seems that current changes in “developmental opportunities” (Thorpe 2007) related to environmental variability are playing a fundamental role in determining the completion of maturation of Atlantic salmon spawning in Norwegian rivers.

## Supporting Information

**Figure S1.** Distribution of weight frequencies by sea age for multiple individuals sampled in 27 Norwegian rivers (*n* = 4062).

**Figure S2.** Time series of Atlantic salmon 1SW proportion from the 59 rivers analyzed. Numbers correspond to “ID” column in Table S1. The proportion of 1SW Atlantic salmon generally decreased with time across the rivers studied. Only 9 of 59 rivers (∼15%) showed an increasing proportion, and the estimated individual long-term trends were not related with latitude or any of a set of river characteristics (Fig. S3). The lower most right panel shows the result of fitting a mixed-effects model of the form . Where Y is the logit-transformed proportion of 1SW fish for each river *i* angled at a year *t*; Year is the smolt year centered by subtracting the mean (1998); *β*_s_ are the fixed effects to be estimated; *a* is the random river (*i*) effect for the intercept assumed to follow a normal distribution with mean zero and variance ; and *ɛ*_*i*,*t*_ is the within-group error term assumed to be first-order autocorrelated (i.e., *ɛ*_*i*,*t*_ = *φɛ*_*i*,*t*-1_ + *η*_*i*,*t*_, where ). In addition, the variance of the residuals was modeled as an exponential function of the fitted values (i.e., , where *δ* is a parameter to be estimated that describes the estimated change in variance with the fitted values (*v*_*i*,*t*_)). Note that a model that included a random effect on the slope was not more optimal than the random intercept model according to BIC. The red line represents the fitted values for the population of rivers and is specified by the following equation , where both coefficients are statistically significant (*P* < 0.0001). Whereas the gray lines represent the within-group fitted curves. The random effects were as follows: *a*_*i*_ SD () = 0.797 (95% CI: 0.659; 0.964), and residual SD (*σ*^2^) = 0.491 (95% CI: 0.444; 0.545). Finally, *φ*_1_ = −0.18 (95% CI: –0.25; –0.11), and *δ* = 0.22 (95% CI: 0.16; 0.28). Overall, the predicted odds of 1SW fish for the whole population of rivers studied decreased by a factor of 0.96 per year (i.e., a 4%/y) during the smolt year classes from 1991 to 2005.

**Figure S3.** Scatterplots of all smolt year coefficient (i.e., the time trend obtained from fitting linear models to each river's 1SW logit-transformed proportion as shown in Figure S2) versus latitude (a) and various habitat characteristics obtained from L'Abée-Lund et al. (2004), (b) altitude (m above sea level), (c) river steepness (m/km), (d) catchment area (km^2^), (e) river length (km), and (f) coastal distance (km).

**Figure S4.** Partial plots showing the relationship between 1SW fish proportion and the PC1 of SST in September (a) and ln-transformed discharge (b). The rugs show the distribution of the data. These plots were obtained using the “mgcv 1.7–13” package (Wood 2006) after fitting a generalized additive mixed model to the data using the same model structure as depicted in Table 1.

**Figure S5.** Optimal model validation. (a) Normality of the within-group errors, (b) scatterplot of the standardized within-group residuals versus the within-group fitted values, (c) normal plot of estimated random effects, and (d) scatterplot of observed versus fitted values. Note that temporal autocorrelation in the normalized residuals in each river was not apparent. In addition, fitting variograms to the normalized residuals of the optimal model per year did not show any remaining spatial correlation either.

**Table S1.** List of the rivers which interannual 1SW and 2SW numbers of fish were analyzed in this study. Latitude (N) and longitude (E) give the geographical position of each river mouth. Overall mean ± SD for each sea age group is also shown.

**Table S2.** Comparison of different models showing the number of parameters, Bayesian Information Criteria (BIC), and the difference in BIC values between each model and the model with the optimal random structure. First, we tested the appropriateness of random intercept and slopes (models 1–4), then we used different ARMA correlation structures for modeling within-group serial correlation (models 5–7). Finally, we modeled the variance structure as described in the main text (model 8). Models were fitted by using restricted maximum likelihood estimation (REML). Note that SST in May, that is, the first month post-smolts live in seawater, was used here.

**Table S3.** Once the optimal (preliminary) random structure has been found (model 8 in Table S2), the fixed components were selected using maximum likelihood estimation (ML) for meaningful comparisons. That is, sea surface temperature (SST) in May (i.e., first month at sea after seaward migration) was used to fit models in Table S2, however, SST in other months could be more important (see Introduction and Discussion in the main text). Therefore, we ran models using SST from different months. Note, however, that choosing a different month could cause a potential problem related to the importance of the previously selected random structure to include in the final formulation. To make the final analyses more robust we rerun models 1–8 using SST in September (the month providing the optimal fit) finding the same random structure.
