# Supplementary material for: Contemporary ocean warming and freshwater conditions are related to later sea age at maturity in Atlantic salmon spawning in Norwegian rivers
Source: Ecol Evol. 2012 Jul 28;2(9):2192–203. doi: 10.1002/ece3.337 (PMC3488670; doi:10.1002/ece3.337)
Supplement: Supplementary file 1 [file ece30002-2192-SD1.doc]

**Supplementary Material**

**
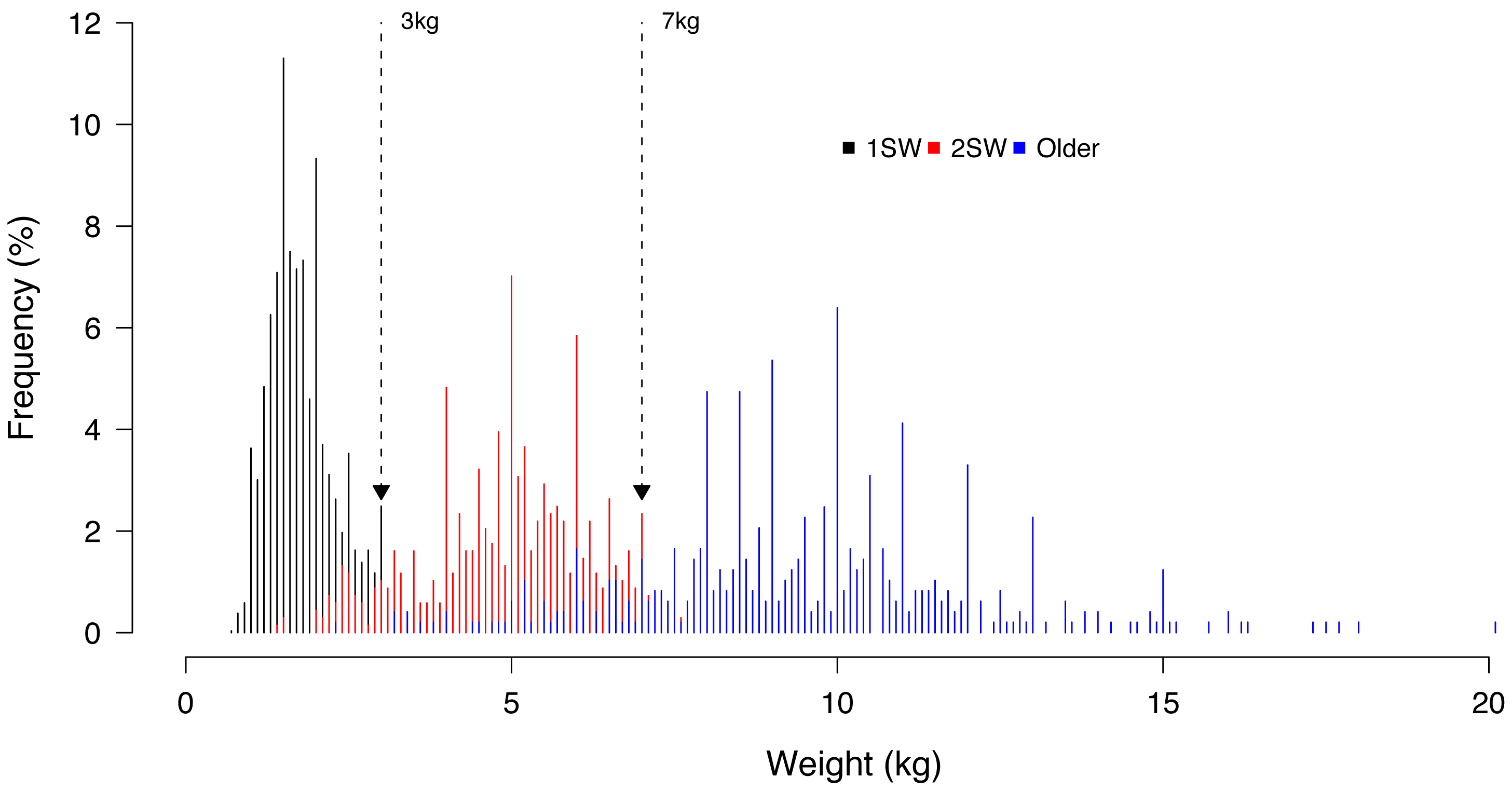
**

**Figure S1.** Distribution of weight frequencies by sea age for multiple individuals sampled in 27 Norwegian rivers (*n* = 4062).

**Table S1.** List of the rivers which interannual 1SW and 2SW numbers of fish were analyzed in this study. Latitude (N) and longitude (E) give the geographical position of each river mouth. Overall mean ± SD for each sea age group is also shown.

| ID | River | Latitude | Longitude | 1SW | | 2SW | |
| --- | --- | --- | --- | --- | --- | --- | --- |
|  |  |  |  | Nº fish | SD | Nº fish | SD |
| 1 | Glomma | 59º12’ | 10º57’ | 96.93 | 36.28 | 86.73 | 37.57 |
| 2 | Drammenselva | 59º44’ | 10º14’ | 913.53 | 299.75 | 673.53 | 322.99 |
| 3 | Numedalslågen | 59º02’ | 10º03’ | 2072.80 | 927.35 | 1736.36 | 505.62 |
| 4 | Skienselva | 59º07’ | 9º37’ | 284.87 | 123.36 | 121.47 | 67.57 |
| 5 | Sokndalselva | 58º19’ | 6º17’ | 527.29 | 357.98 | 126.57 | 75.16 |
| 6 | Ogna | 58º31’ | 5º48’ | 1692.33 | 747.42 | 212.80 | 67.88 |
| 7 | Håelva | 58º41’ | 5º32’ | 2059.73 | 1014.72 | 236.07 | 108.12 |
| 8 | Figgjo | 58º48’ | 5º33’ | 2072.73 | 812.54 | 510.47 | 213.01 |
| 9 | Suldalslågen | 59º29’ | 6º15’ | 213.29 | 129.47 | 140.93 | 121.05 |
| 10 | Etneelva | 59º40’ | 5º56’ | 508.47 | 187.20 | 285.53 | 127.42 |
| 11 | Eikefetelva | 60º43’ | 5º33’ | 133.27 | 82.11 | 24.90 | 22.62 |
| 12 | Gaular | 61º22’ | 5º41’ | 441.07 | 209.02 | 251.13 | 160.71 |
| 13 | Nausta | 61º31’ | 5º44’ | 991.60 | 556.17 | 333.80 | 203.32 |
| 14 | Eidselva | 61º54’ | 5º59’ | 286.47 | 99.49 | 140.20 | 84.94 |
| 15 | Ervikelva | 62º10’ | 5º07’ | 120.40 | 73.02 | 24.80 | 21.66 |
| 16 | Åheimselva | 62º03’ | 5º37’ | 242.66 | 179.19 | 26.52 | 17.84 |
| 17 | Austefjordelva | 62º04’ | 6º19’ | 137.87 | 126.35 | 35.80 | 26.65 |
| 18 | Storelva | 62º08’ | 6º15’ | 175.50 | 114.14 | 72.38 | 84.45 |
| 19 | Ørstaelva | 62º11’ | 6º08’ | 379.38 | 261.42 | 102.18 | 67.47 |
| 20 | Bondalselva | 62º12’ | 6º28’ | 513.49 | 425.90 | 140.93 | 89.10 |
| 21 | Vikelva | 62º06’ | 6º34’ | 125.23 | 84.08 | 33.49 | 16.81 |
| 22 | Velledalselva | 62º20’ | 6º36’ | 526.73 | 371.49 | 101.33 | 45.15 |
| 23 | Strandaelva | 62º18’ | 6º56’ | 487.96 | 385.43 | 95.72 | 43.60 |
| 24 | Korsbrekkelva | 62º05’ | 6º52’ | 197.60 | 114.52 | 77.67 | 48.44 |
| 25 | Stordalselva | 62º27’ | 6º59’ | 275.93 | 155.68 | 112.47 | 62.10 |
| 26 | Oselva | 62º48’ | 7º43’ | 481.03 | 316.65 | 37.99 | 22.74 |
| 27 | Sylteelva | 62º50’ | 7º12’ | 365.05 | 231.25 | 23.12 | 17.35 |
| 28 | Søya | 62º53’ | 8º32’ | 185.36 | 73.35 | 15.21 | 8.61 |
| 29 | Surna | 62º58’ | 8º40’ | 745.20 | 321.97 | 454.73 | 185.20 |
| 30 | Orkla | 63º19’ | 9º50’ | 2270.53 | 1494.54 | 1280.73 | 880.67 |
| 31 | Gaula | 63º21’ | 10º14’ | 3127.07 | 2043.69 | 1923.93 | 1297.12 |
| 32 | Nidelva | 63º26’ | 10º24’ | 690.80 | 539.59 | 336.80 | 248.21 |
| 33 | Stjørdalselva | 63º26’ | 10º54’ | 1212.87 | 651.88 | 521.33 | 301.85 |
| 34 | Verdalsvassdraget | 63º48’ | 11º28’ | 794.40 | 479.79 | 349.73 | 234.45 |
| 35 | Skauga | 63º36’ | 9º56’ | 846.33 | 662.42 | 49.93 | 54.24 |
| 36 | Nordelva | 63º46’ | 10º07’ | 401.78 | 369.32 | 25.19 | 21.89 |
| 37 | Stordalselva | 63º57’ | 10º13’ | 3174.93 | 1677.45 | 275.00 | 129.34 |
| 38 | Steinselva | 64º18’ | 10º31’ | 851.07 | 559.48 | 59.25 | 62.35 |
| 39 | Årgårdselva | 64º18’ | 11º12’ | 3430.20 | 2051.61 | 90.67 | 36.40 |
| 40 | Namsen | 64º28’ | 11º35’ | 4393.13 | 1591.12 | 1813.47 | 692.59 |
| 41 | Salvassdraget | 64º42’ | 11º26’ | 487.60 | 159.24 | 82.87 | 34.96 |
| 42 | Kongsmoelva | 64º53’ | 12º27’ | 128.20 | 65.80 | 18.33 | 14.46 |
| 43 | Åelva | 65º05’ | 12º27’ | 264.93 | 163.42 | 58.07 | 36.56 |
| 44 | Vefsna | 65º50’ | 13º13’ | 357.07 | 152.82 | 207.13 | 110.19 |
| 45 | Ranavassdraget | 66º20’ | 14º09’ | 235.80 | 177.17 | 165.27 | 131.38 |
| 46 | Laukhellevassdraget | 69º14’ | 17º51’ | 407.53 | 223.38 | 94.33 | 52.19 |
| 47 | Målselva | 69º14’ | 18º31’ | 1033.93 | 468.59 | 383.87 | 269.22 |
| 48 | Altaelva | 69º58’ | 23º23’ | 1747.27 | 673.17 | 389.73 | 184.56 |
| 49 | Repparfjordelva | 70º27’ | 24º20’ | 1819.14 | 814.87 | 242.79 | 144.62 |
| 50 | Stabburselva | 70º11’ | 24º54’ | 570.93 | 270.69 | 85.07 | 49.89 |
| 51 | Lakselva | 70º04’ | 24º55’ | 487.34 | 129.42 | 159.58 | 70.89 |
| 52 | Børselva | 70º18’ | 25º32’ | 670.20 | 276.19 | 155.60 | 94.27 |
| 53 | Langfjordelva | 70º37’ | 27º36’ | 555.60 | 252.51 | 99.80 | 65.30 |
| 54 | Tanaelva | 70º28’ | 28º20’ | 12102.80 | 5619.22 | 3994.07 | 2171.87 |
| 55 | Vesterelva | 70º32’ | 29º58’ | 240.07 | 289.98 | 98.73 | 154.77 |
| 56 | Komagelva | 70º14’ | 30º32’ | 528.53 | 254.39 | 205.47 | 204.45 |
| 57 | Munkelva | 69º39’ | 29º27’ | 113.87 | 46.00 | 10.98 | 7.61 |
| 58 | Neidenelva | 69º42’ | 29º24’ | 1970.86 | 557.45 | 378.60 | 199.22 |
| 59 | Karpelva | 69º40’ | 30º23’ | 138.12 | 53.83 | 6.53 | 3.93 |


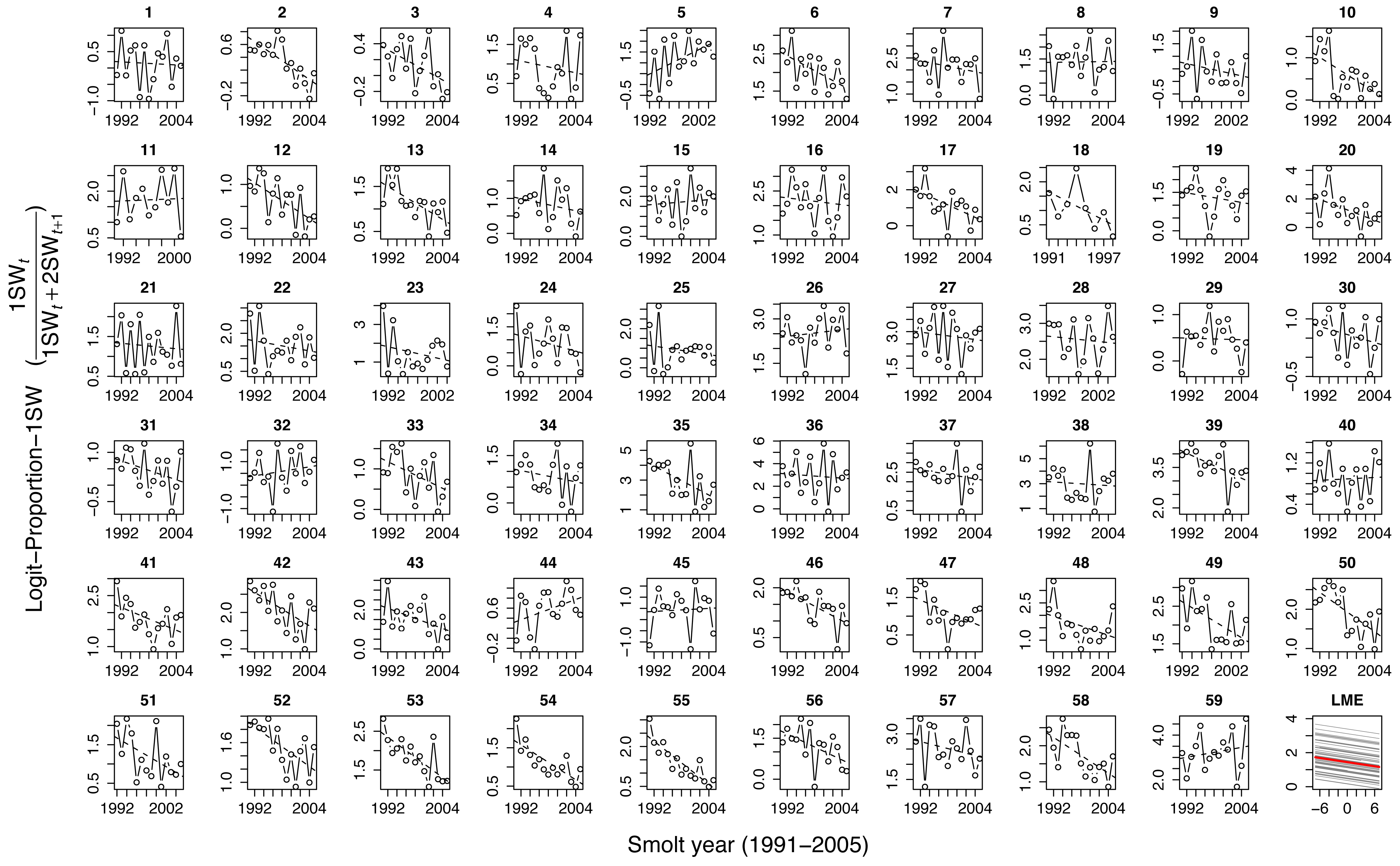


**Figure S2.** Time series of Atlantic salmon 1SW proportion from the 59 rivers analyzed. Numbers correspond to ‘ID’ column in Table S1. The proportion of 1SW Atlantic salmon generally decreased with time across the rivers studied. Only 9 out of 59 rivers (~15%) showed an increasing proportion and the estimated individual long-term trends were not related with latitude or any of a set of river characteristics (Fig. S3). The lower most right panel shows the result of fitting a mixed-effects model of the form . Where Y is the logit-transformed proportion of 1SW fish for each river *i* angled at a year *t*; Year is the smolt year centered by subtracting the mean (1998); *β*_s_ are the fixed effects to be estimated; *a* is the random river (*i*) effect for the intercept assumed to follow a normal distribution with mean zero and variance $s_{a}^{2}$; and $e_{i,t}$ is the within-group error term assumed to be first-order autocorrelated (i.e., , where ). In addition, the variance of the residuals was modeled as an exponential function of the fitted values (i.e. , where *δ* is a parameter to be estimated that describes the estimated change in variance with the fitted values ()). Note that a model that included a random effect on the slope was not more optimal than the random intercept model according to BIC. The red line represents the fitted values for the population of rivers and is specified by the following equation where both coefficients are statistically significant (*P* < 0.0001). Whereas the grey lines represent the within-group fitted curves. The random effects were as follows: *a_i_* SD ($s_{a}^{2}$) = 0.797 (95% CI: 0.659; 0.964), and residual SD ($s^{2}$) = 0.491 (95% CI: 0.444; 0.545). Finally, (95% CI: –0.25; –0.11), and *δ* = 0.22 (95% CI: 0.16; 0.28). Overall, the predicted odds of 1SW fish for the whole population of rivers studied decreased by a factor of 0.96 per year (i.e. a 4% y^–1^) during the smolt year classes from 1991 to 2005.


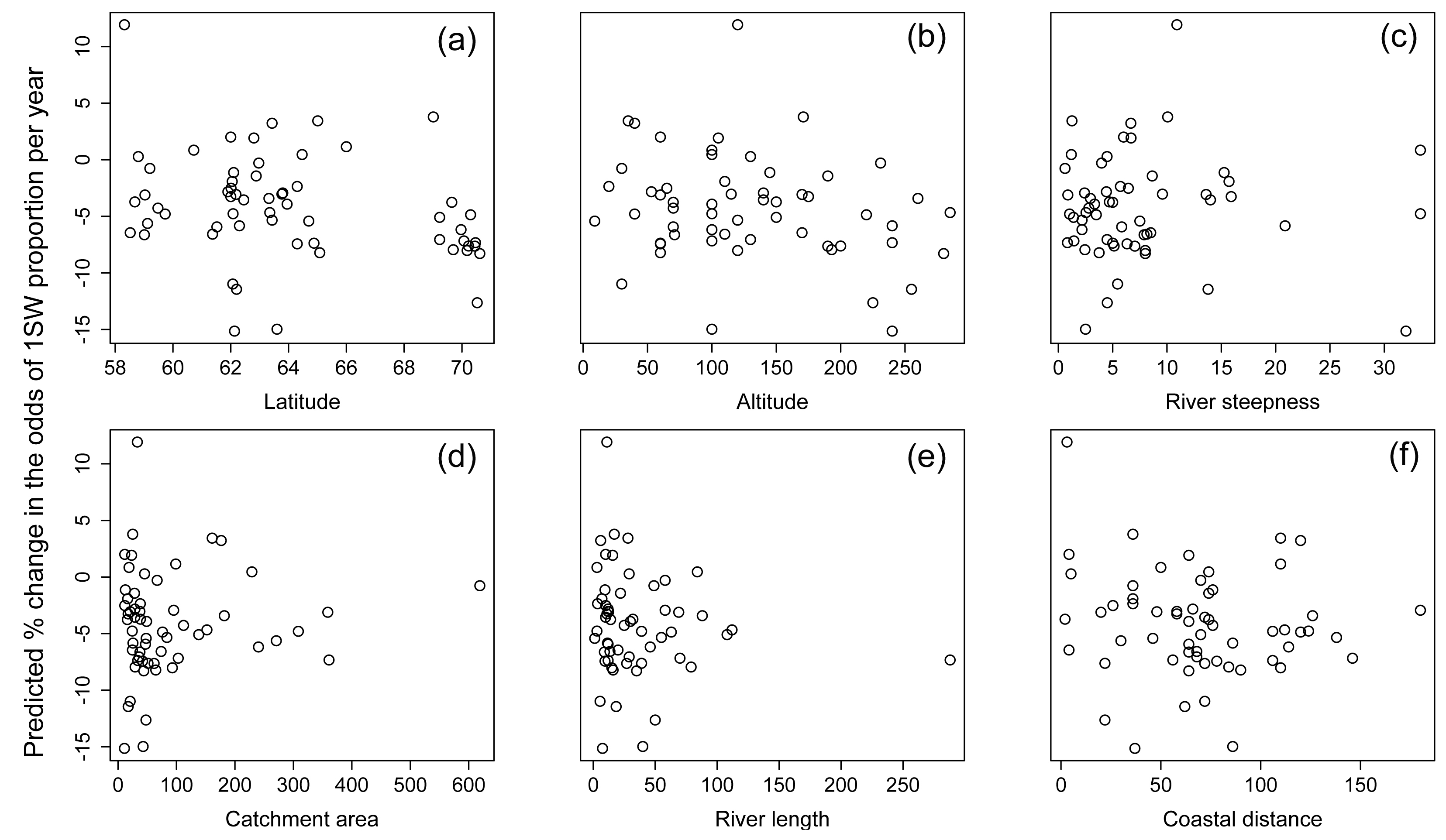


**Figure S3.** Scatterplots of all smolt year coefficient (i.e. the time trend obtained from fitting linear models to each river’s 1SW logit-transformed proportion as shown in Figure S2) versus latitude (a) and various habitat characteristics obtained from L’Abée-Lund et al. (2004): (b) altitude (m above sea level), (c) river steepness (m km^–1^), (d) catchment area (km^2^), (e) river length (km), and (f) coastal distance (km).

**Reference**

L’Abée-Lund, J. H., L. A. Vøllestad, and S. Beldring. 2004. Spatial and temporal variation in the grilse proportion of Atlantic salmon in Norwegian rivers. Transactions of the American Fisheries Society 133:743–761.

**Table S2.** Comparison of different models showing the number of parameters, Bayesian Information Criteria (BIC) and the difference in BIC values between each model and the model with the optimal random structure. First, we tested the appropriateness of random intercept and slopes (models 1 to 4), then, we used different ARMA correlation structures for modeling within-group serial correlation (models 5 to 7). Finally, we modeled the variance structure as described in the main text (model 8). Models were fitted by using restricted maximum likelihood estimation (REML). Note that SST in May, that is, the first month post-smolts live in seawater, was used here.

| Model nº | Random effects | Correlation structure | Parameters | BIC | ΔBIC |
| --- | --- | --- | --- | --- | --- |
| 1 | none | none | 4 | 2402.47 | 378.61 |
| 2 | *β*_0_ | none | 5 | 2079.72 | 55.86 |
| 3 | *β*_0_ and *β*_1_ | none | 7 | 2092.68 | 68.81 |
| 4 | *β*_0_ and *β*_2_ | none | 7 | 2084.49 | 60.63 |
| 5 | *β*_0_ | ARMA(1,0) | 6 | 2070.57 | 46.71 |
| 6 | *β*_0_ | ARMA(0,1) | 6 | 2073.82 | 49.96 |
| 7 | *β*_0_ | ARMA(1,1) | 7 | 2074.61 | 50.75 |
| 8 | *β*_0_ | ARMA(1,0) | 7 | 2023.86 | 0 |

**Table S3.** Once the optimal (preliminary) random structure has been found (model 8 in Table S2), the fixed components were selected using maximum likelihood estimation (ML) for meaningful comparisons. That is, sea surface temperature (SST) in May (i.e., first month at sea after seaward migration) was used to fit models in Table S2, however, SST in other months could be more important (see Introduction and Discussion in the main text). Therefore, we ran models using SST from different months. Note, however, that choosing a different month could cause a potential problem related to the importance of the previously selected random structure to include in the final formulation. To make the final analyses more robust we re-run models 1 to 8 using SST in September (the month providing the optimal fit) finding the same random structure.

| Model nº | Model structure | BIC | ΔBIC |
| --- | --- | --- | --- |
| 9 |  | 2004.77 | 29.50 |
| 10 |  | 2026.48 | 51.22 |
| 11 |  | 2010.62 | 35.35 |
| 12 |  | 2010.24 | 34.97 |
| 13 |  | 1975.27 | 0 |
| 14 |  | 1992.28 | 17.02 |
| 15 |  | 1997.16 | 21.89 |
| 16 |  | 2010.13 | 34.87 |
| 17 |  | 2000.33 | 25.07 |
| 18 |  | 1993.13 | 17.86 |
| 19 |  | 1988.48 | 13.21 |
| 20 |  | 1985.39 | 10.13 |


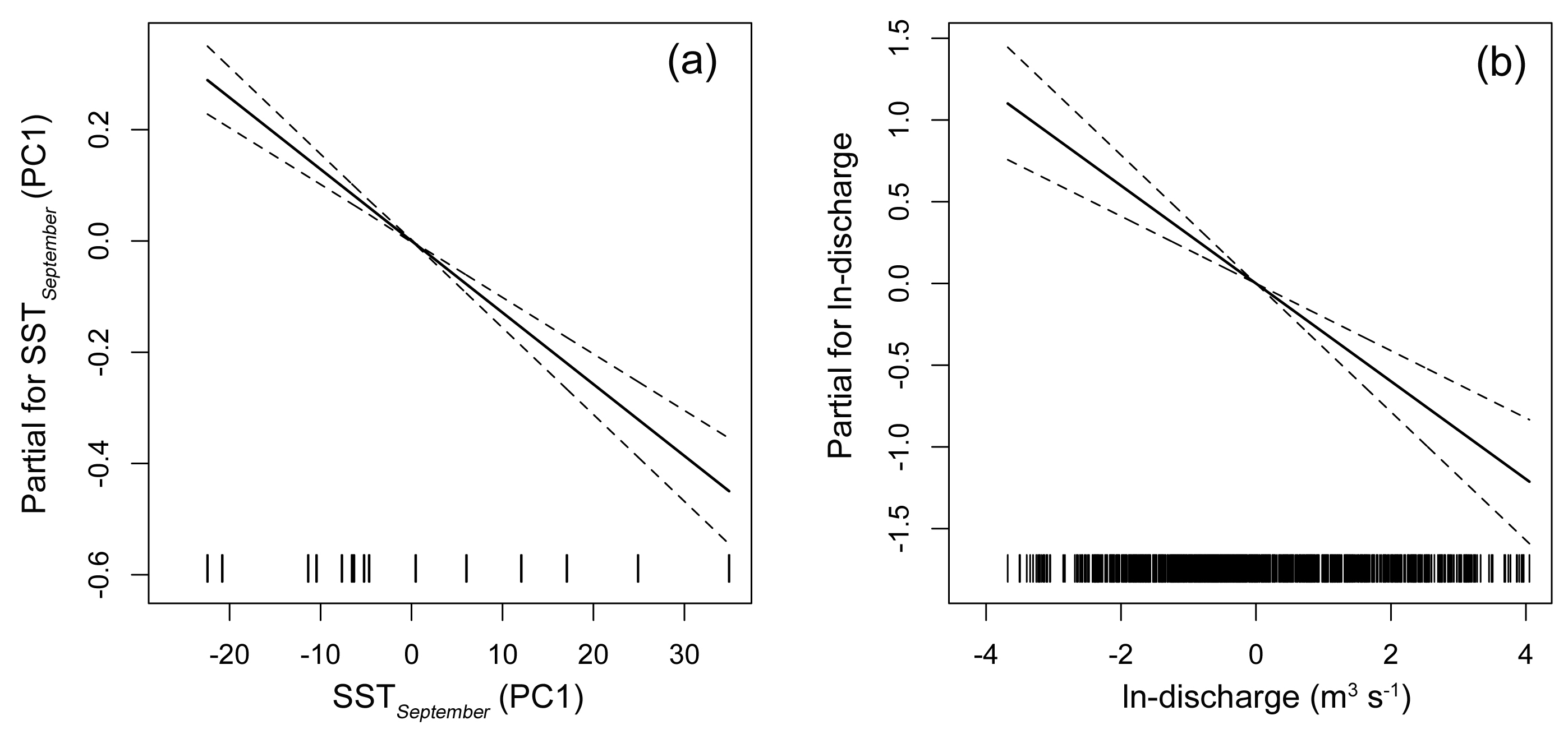


**Figure S4.** Partial plots showing the relationship between 1SW fish proportion and the PC1 of SST in September (a), and ln-transformed discharge (b). The rugs show the distribution of the data. These plots were obtained using the “mgcv 1.7-13” package (Wood 2006) after fitting a generalized additive mixed model to the data using the same model structure as depicted in Table 1.

**Reference**

Wood, S. 2006. Generalized Additive Models: An Introduction with R. Chapman & Hall, London, UK. 391 pp.


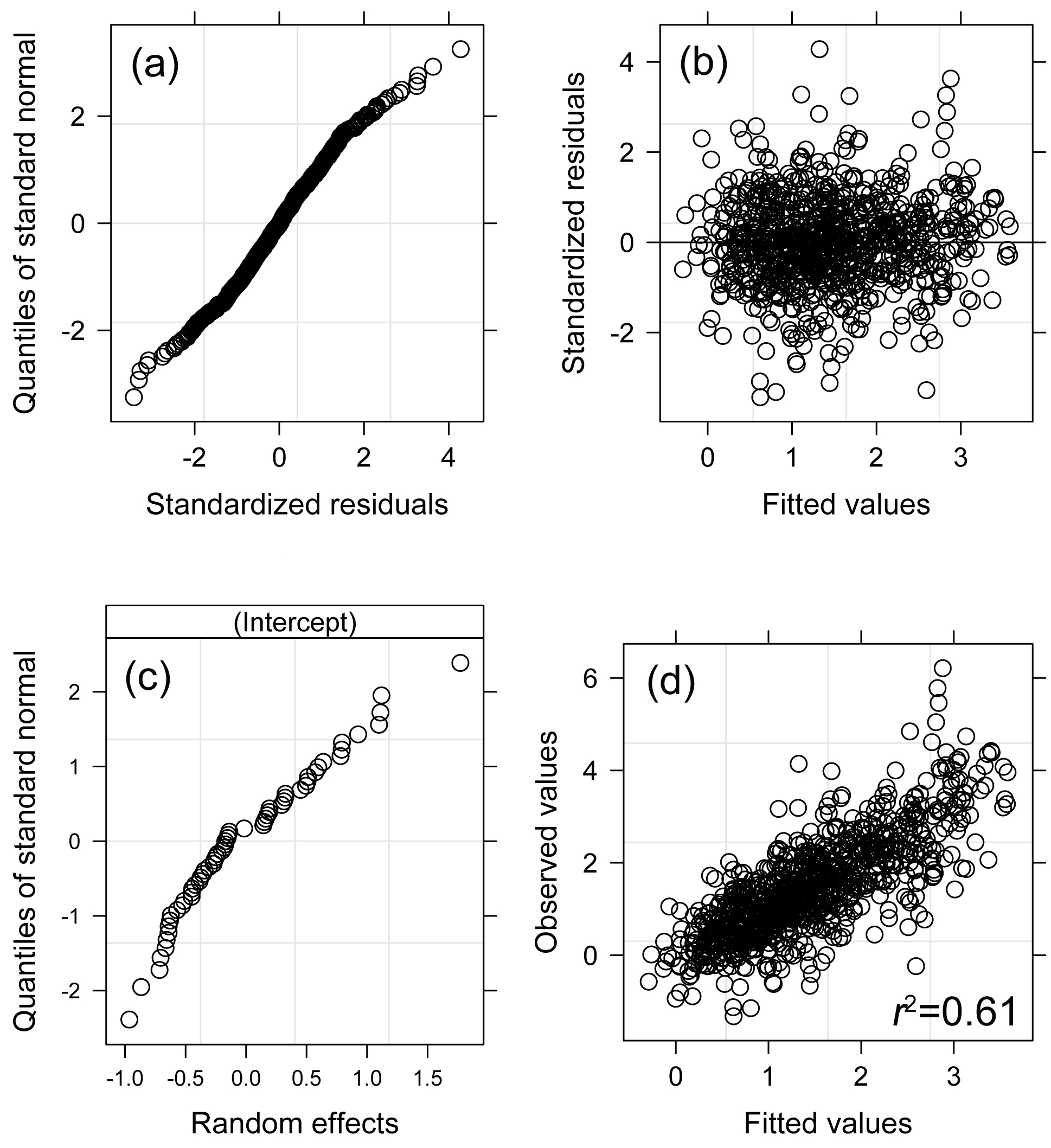


**Figure S5.** Optimal model validation. (a) Normality of the within group errors, (b) scatterplot of the standardized within group residuals versus the within group fitted values, (c) normal plot of estimated random effects, and (d) scatterplot of observed versus fitted values. Note that temporal autocorrelation in the normalized residuals in each river was not apparent. In addition, fitting variograms to the normalized residuals of the optimal model per year did not show any remaining spatial correlation either.
